# Understanding analytical systems technology for selective analysis

**DOI:** 10.1155/S146392468800032X

**Published:** 1988

**Authors:** T. D. Geary

**Affiliations:** Institute of Medical and Veterinary Science South Australia Adelaide Australia


					Journal of Automatic Chemistry Vol. 10, No. 4 (October-December 1988), p. 163
Special Section:
Understanding analytical systems
technology for selective analysis
Introduction
T. D. Geary
Institute ofMedical and Veterinary Science, Adelaide, South Australia, Australia
The members ofthe International Federation ofClinical Chemistry Expert Panel on Instrumentation made a decision to
present a series of symposia to discuss and allay fears caused by the advances in technology which have led to the
development of the 'black box'.
The Organizing Committee ofthe International Congress ofClinical Chemistry (The Hague, 1987) was approached and
time was allocated for the first ofthese symposia. The symposium was structured to explain in detail the functions of the
'black box', as well as its components, and had the appropriate title Technologyfor Selective Analysis. Selective analysers
embody many ofthe components which contribute to the feeling ofuncertainty, referred to as the 'black box syndrome'.
The 'black box' can be divided into its individual components as indicated for an analytical instrument in figure 1. It is
important to be conversant with the operation of the individual parts in order to understand the functioning ofthe total.
SAMPLING
MIXING
INCUBATING ANALYTICAL
MEASURING INSTRUMENT
CALCULATING
Figure 1. DISPLAYING
Before this task can be tackled it is necessary to provide some relevant definitions. The first ofthese being the definition of
an analytical instrument, which is a device or a combination ofdevices used to carry out an analytical process. This is
illustrated in figure 1. The second is an analytical system which is a system ofdevices with process control. Reagents are
considered to be an integral part of an analytical system (figure 2)"
ANALYTICAL INSTRUMENT
figlrg 2. REAGENTS
ANALYTICAL
SYSTEM
The development of the Skeggs analytical instrument was a significant advance in clinical laboratory medicine. It
brought together mixing, sampling, separating, measuring and displaying devices in a sequence which mimicked the
manual operation in a manner which provided increased sampling rate and led to decrease in the imprecision of the
analytical methods in use. The instrument relied upon continuous flow air segmentation to transport the reaction
mixture. Since that time, there have been other transport systems developed, which have provided opportunities in other
areas ofthe clinical laboratory. These innovations have led to the systems being more operator independent- but with an
increase in complexity. This increase in complexity and lack of involvement in the assay contributes to the worries
associated with the 'black box'.
The symposium considered the following topics"
(1) General principles for the classification of analysers.
(2) Selectivity and random access in automatic analysers.
(3) System control: sampling, diluting, dispensing, and temperature control.
(4) System control: optics and electronic system control.
(5) Transport and carry-over.
(6) Organizational aspects of selective analysers; choosing an instrument appropriate to the laboratory's
requirements.
163

				

